# Real-time infection prediction with wearable physiological monitoring and AI to aid military workforce readiness during COVID-19

**DOI:** 10.1038/s41598-022-07764-6

**Published:** 2022-03-08

**Authors:** Bryan Conroy, Ikaro Silva, Golbarg Mehraei, Robert Damiano, Brian Gross, Emmanuele Salvati, Ting Feng, Jeffrey Schneider, Niels Olson, Anne G. Rizzo, Catherine M. Curtin, Joseph Frassica, Daniel C. McFarlane

**Affiliations:** 1 Philips Research North America, Cambridge, MA USA; 2 Defense Innovation Unit, Mountain View, CA USA; 3 The Guthrie Clinic, Sayre, PA USA; 4 Department of Surgery, Palo Alto Veteran Affairs Healthcare System, Palo Alto, CA USA; 5 Institute for Medical Engineering and Science, Massachusetts Institute of Technology, Cambridge, MA USA

**Keywords:** Infectious diseases, Machine learning

## Abstract

Infectious threats, like the COVID-19 pandemic, hinder maintenance of a productive and healthy workforce. If subtle physiological changes precede overt illness, then proactive isolation and testing can reduce labor force impacts. This study hypothesized that an early infection warning service based on wearable physiological monitoring and predictive models created with machine learning could be developed and deployed. We developed a prototype tool, first deployed June 23, 2020, that delivered continuously updated scores of infection risk for SARS-CoV-2 through April 8, 2021. Data were acquired from 9381 United States Department of Defense (US DoD) personnel wearing Garmin and Oura devices, totaling 599,174 user-days of service and 201 million hours of data. There were 491 COVID-19 positive cases. A predictive algorithm identified infection before diagnostic testing with an AUC of 0.82. Barriers to implementation included adequate data capture (at least 48% data was needed) and delays in data transmission. We observe increased risk scores as early as 6 days prior to diagnostic testing (2.3 days average). This study showed feasibility of a real-time risk prediction score to minimize workforce impacts of infection.

## Introduction

Global threats to public health^1^, like the ongoing COVID-19 pandemic, complicate efforts to maintain workforce productivity impacting commerce and national security^2^. One study estimated the disruption of the COVID-19 pandemic to the United States (US) economy at $16 trillion (about 90% of the annual US gross domestic product (GDP))^3^. These costs included: $7.6 trillion in direct losses to GDP; $4.4 trillion for premature death; $2.6 trillion for long-term health impairment; and $1.6 trillion for mental health impairment^3^. The negative effects on workforces have been widely reported^4,5^, including hardships for: global economies^6^, businesses^7–9^, agriculture^10^, rural economies^11^, mental health in the workplace^12^, healthcare^13^, and worsened domestic violence^14^.

The risk to workforce productivity from infection is especially troublesome for those organizations responsible for providing essential services. During the current pandemic, staffing was further complicated by a high percentage of mildly symptomatic and pre-symptomatic infected individuals with sufficient viral load to unknowingly infect others in their community^15–17^. The US Department of Defense (DoD) is an essential service with commitments to be ‘on location,’ and ‘in person,’ during public health crises^18^. The US DoD invests heavily in maintaining the readiness of its workforce to conduct essential missions^19–21^. The risk posed by infectious diseases has long been a concern for military operations^22^. During public health crises, the US DoD uses multiple methods to collect information about the health of servicemembers, including: surveys^23^, formal readiness reporting^21^, and frequent monitoring and testing^24^.

The present article reports on US DoD-sponsored research to investigate use of wearable physiological monitoring for early prediction of infection and real-time notification of potential exposures via predictive machine learning. It is hypothesized that a physiologically-based informatics solution is possible, and that delivery of an early warning "check engine light" service could alert organizations and individuals of possible early cases of infection. Prior work has shown the potential to predict COVID-19 using a variety of wearables^25–31^. Supplementary Table 1 contrasts this approach with other common methods and highlights the potential superiority for enabling early and proactive action to minimize exponential outbreaks. This approach could deliver a powerful new practical tool to combat global threats to workforce readiness from infectious diseases.

A large prospective study was conducted to explore the use of wearables for early detection of COVID-19 in an active workforce. This study utilized multiple commercially off-the-shelf (COTS) wearable devices to deliver a real-time service for inference of infection risk with US military personnel working during the COVID-19 pandemic. The goal of this effort was to rapidly operationalize the Rapid Analysis of Threat Exposure (RATE) algorithm^32^, which was originally developed by Philips under a Defense Threat Reduction Agency (DTRA) and Defense Innovation Unit (DIU)-sponsored program (2018–2019) to predict hospital-acquired infection. It was hypothesized that RATE could deliver a pre-symptomatic early warning service for exposure to SARS-CoV-2 to support and maintain mission readiness of healthcare personnel and critical DoD staff during the COVID-19 pandemic. The initial version of this real-time prototype system with continual wearable physiological monitoring was first deployed with active military users on June 23, 2020. The study progressed through intense co-creation with the US military to maintain constant grounding with the needs of mobile workers and iteratively improve the technical maturity and usability (the main objectives) and service utility. The resulting evidence supports an assertion of the feasibility of the proposed technology.

### Declarations

The field study reported here was performed in accordance with relevant guidelines and regulations, including receiving preapproval for all protocols by institutional review boards: Clinical Investigation Department Naval Medical Center, San Diego CIP #NMCSD.2020.0027; Air Force Research Laboratory Wright-Patterson Air Force Base, Ohio #WR20200175H; and Stanford University Investigational Review Board, Stanford CA, eProtocol#55805. Informed consent was obtained from all participants.

## Methods

The methods are divided into three parts: (1) the Study Description section details the recruitment and data collection study; (2) the Study Platform section describes the platform developed for data collection and infection prediction; and (3) the Predictive Model Training section describes the machine learning pipeline developed for training an effective predictive model of COVID-19.

### Study description

Participants were active military personnel recruited from multiple US DoD sites.

The original RATE research^32^ informed the selection of wearable devices for this study: the Garmin watch (Fenix 6 and Vivoactive 4), Oura Ring, and Empatica E4 wristband. These devices can measure several physiological variables including heart rate (HR), inter-beat interval (IBI), respiration rate (RR), pulse oxymetry, skin temperature, and accelerometer data (see Supplementary Table 2 for a breakdown by device). For purposes of subsequent data analyses, Empatica data (including photoplethysmography (PPG) and galvanic skin response (GSR)) were excluded due to the small number of participants assigned this device.

Study participants were distributed a Garmin watch (Fenix 6 or Vivoactive 4 models) and an Oura ring. Oura sizing kits were used to properly fit the ring based on participant preference. No modifications to the manufacturers’ labeling or ‘instructions for use’ were made for the purpose of this study, and participants initialized and configured their devices per the respective wearable manufacturer’s instructions. Almost all participants (98.6%) wore Garmin smartwatches, and 89.8% wore Oura smart rings (with 88.4% wearing both). Participants were asked to wear the device(s) continuously for the duration of the study, except during battery recharging, when the subject’s job required removing the wearable(s) to perform a job function, or whenever the participant was uncomfortable wearing the device.

The predictive service was delivered to all through web UIs. The study platform was designed to avoid collection of any trackable location or personally identifiable information (PII) at any time, even during enrollment. This was achieved through a tight collaboration with the DoD and a unique one-time registration process. Randomly-generated login credentials were shared with principal investigators, who distributed these to participants for the purpose of accessing the study website to pair their wearable devices with the study. During this one-time registration, basic demographic categories were collected for: age, height, weight, and pre-existing medical conditions. From that point forward, wearable data uploaded to the manufacturer’s cloud via the respective wearable vendor app was made accessible to the study platform for calculating and continuously updating an infection risk score.

In addition to wearing the devices, participants were asked to complete a brief daily survey, which collected self-reported symptoms, over-the-counter medications taken, and fiducial points for vaccination or positive test results for infection, including COVID-19 (see Supplementary Table 4 for the list of survey questions). Individuals were tested by a COVID-19 RT-PCR or Rapid test through either military facilities or civilian test locations. Testing was conducted pursuant to protocols in place at the time and for the location (I.e., the subject presented with symptoms or required for screening and surveillance). Individuals self-reported test date, results date, and results (positive or negative) via the daily survey. The survey data was periodically queried, and data verified with the individual through the site study personnel for all COVID positive cases to ensure the test and symptom fiducial points were accurate. In rare cases, the Site Personnel updated missing or inaccurate the information.

### Study platform description

The platform for data collection and prediction of infection was comprised of four components: (1) a wearable data ingestion module that interfaces with COTS wearable vendors to collect and convert physiological data into a standardized format; (2) a runtime execution environment that implements the predictive model of infection and is asynchronously triggered by the incoming data from '1'; (3) a web-based UI that displays the resulting infection risk score and collects daily surveys for the purpose of generating ground-truth labels for retrospective machine learning; and (4) an offline research environment that allows data scientists to adapt the models based on incremental data acquired over the course of the study. Note that while the focus of this article is on ground-truth labels for COVID-19 infection, the platform readily extends to other applications.

#### COTS wearable device integration

Each of the wearable device vendors has a different secure protocol for data sharing, see Supplementary Table 3 for details. Incoming data from the devices were batch processed at 10-min intervals to convert the device-specific data into a compressed Python Pandas data frame format that standardized column names for all physiological measurements across devices (see Supplementary Table 6). In addition, the standardization step extracted and persisted pertinent meta-data to a database for efficient querying and retrieval, and to instantiate a real-time pipeline trigger that coordinates the extraction of features from the data to update the time-based state model that is used by the infection prediction model. Data provenance is preserved with respect to the source device, which allows the client algorithms, for example, to query and load heart rate measurements ("hr") from any device using a common data object (Pandas Data Frame) and with simple consistent syntax. The standardization step was implemented via Python objects specific for each device that understood its device's incoming data format. Thus the system was designed to scale to new devices provided a Python object specific to the new device is implemented.

#### Runtime execution environment

**Figure 1:**
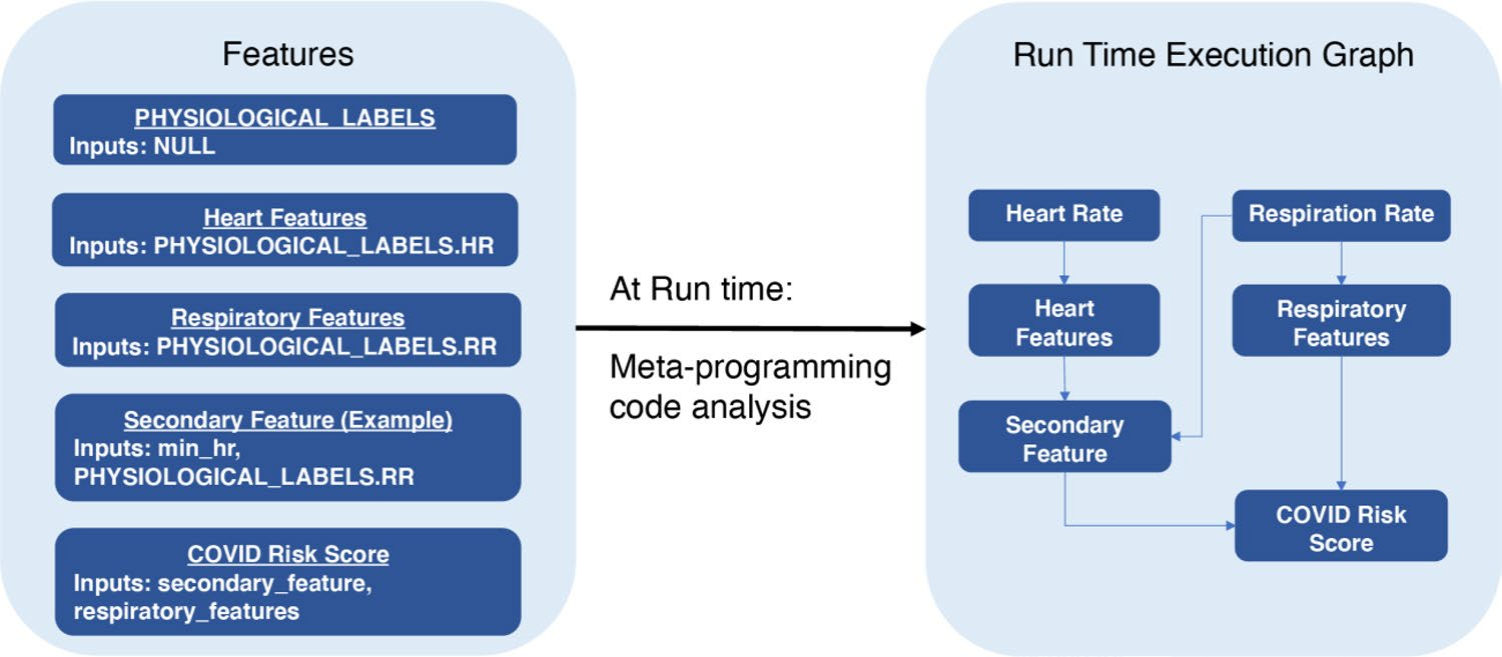
Toy example of an execution graph calculated at runtime. The PHYSIOLOGICAL_LABELS feature represents the standardized wearables data inputs and acts as source nodes in the DAG.

**Figure 2:**
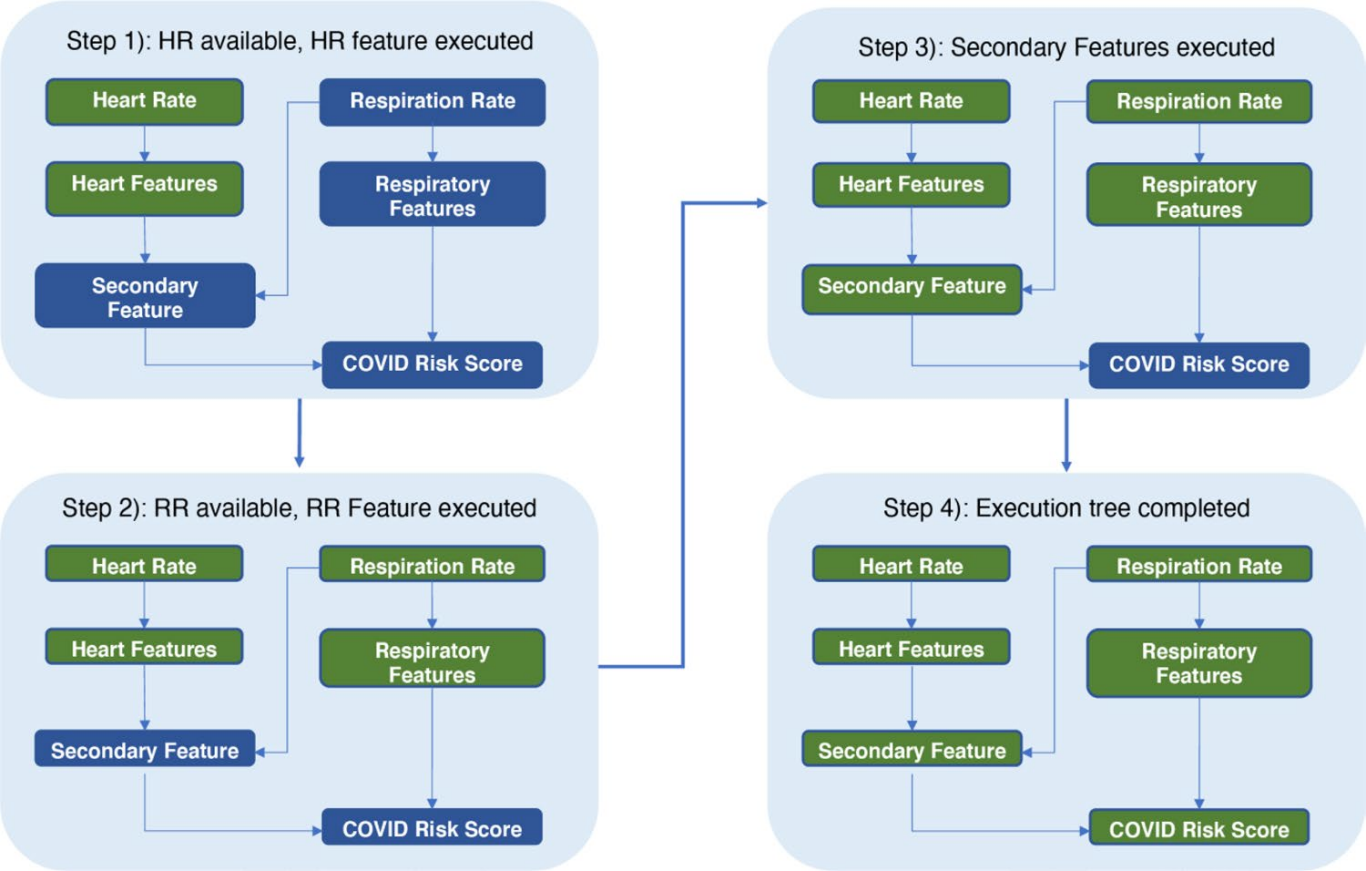
Toy example of the feature pipeline orchestrated by the triggering mechanism on the runtime execution DAG. The trigger maintains the state of the execution graph and its requirements at each step of the process. In this example, the machine learning prediction (COVID risk score) gets first reported as soon as the respiratory feature becomes available. As additional features (e.g., those from heart rate) become available, the prediction is updated with the new information.

The end-to-end pipeline to generate a predictive infection score was subdivided into a set of modular units (also called features), each of which was implemented in Python and performed signal processing and/or prediction tasks based on the input from one or more physiological data feeds. Modular units could also feed as input to other modular units, resulting in a hierarchy of processing that culminated in the calculation of an infection prediction score. At runtime, these units were combined into a directed acyclic graph (DAG) based on input/output relations, which defines the sequential ordering of execution. This architecture enabled several efficiencies based on the specifics of the application: (1) modular units were triggered as soon as their input dependencies became available, which mitigated latencies that may arise due to the asynchronous arrival of data from the wearable vendors; and (2) intermediate outputs in the processing DAG were cached to avoid recalculation. Figure 1 provides a toy example of features defined in the pipeline, and Fig. 2 shows the resulting DAG based on processing the input/output relations from the meta-data at runtime. Note that the deployed machine learning algorithm (COVID Risk Score) is treated as a standard feature in the pipeline. For more specifics on the set of features extracted for the COVID prediction model, see the “Predictive Model Training Applied to COVID-19” section below.

#### Web-based user interface

**Figure 3:**
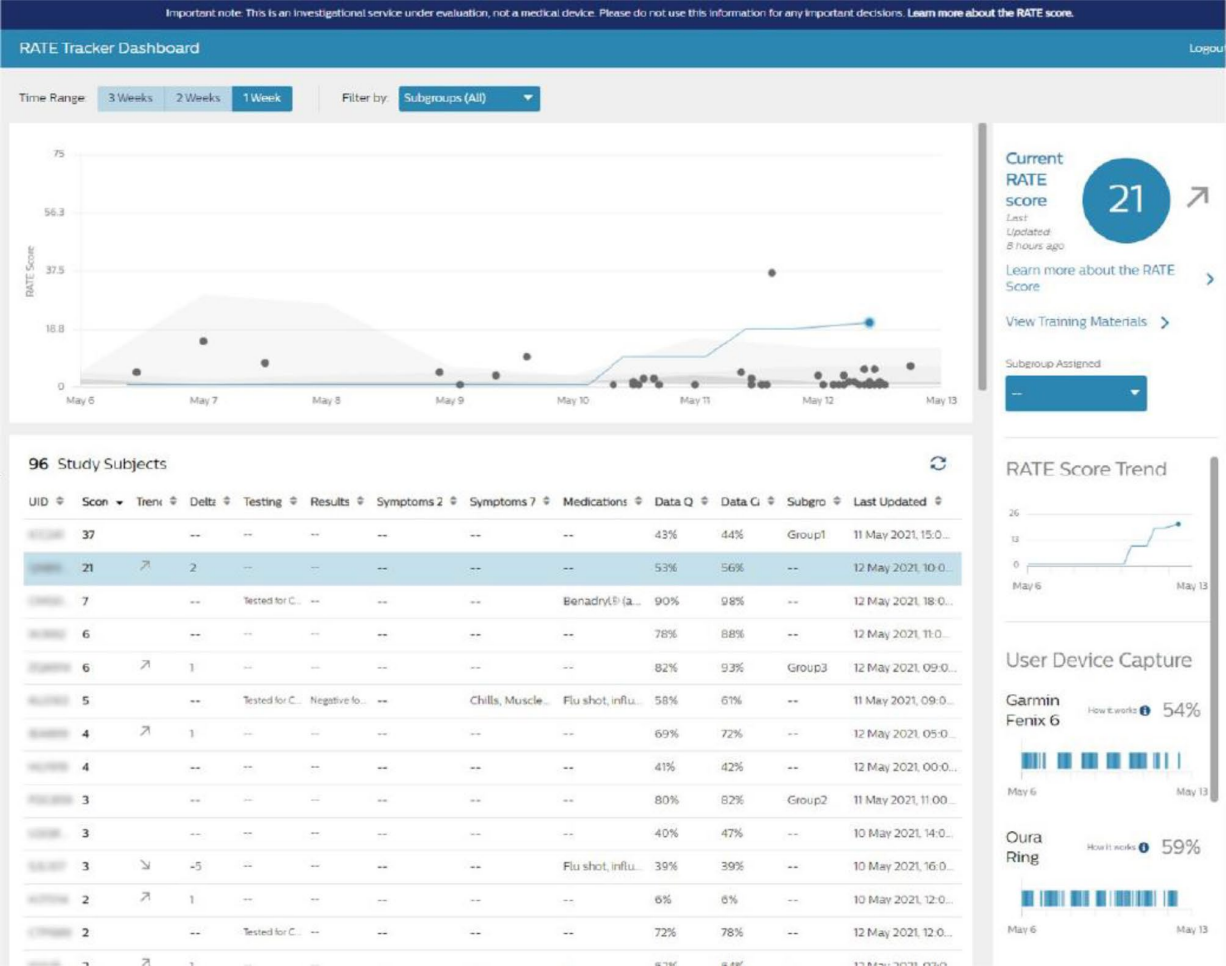
The admin UI allows site coordinators to monitor the infection risk at a site level. Selecting an individual on the scatter plot shows the infection score trend for that user.

Two different web-based user interfaces (UI) were developed to facilitate interactions with the principal investigators, site coordinators, and study participants. The study participants interacted with the individual’s UI (Rate Tracker), while site coordinators, and principal investigators interacted with a subgroup view (Rate Tracker Dashboard). On a daily basis, the study participant log into the UI and are prompted to submit their surveys, after which they are re-directed to an individual dashboard that displayed the current infection risk score and weekly trend, wearable device(s) data capture quality, and summary of survey responses. To assist with wearable device integration issues, links to troubleshooting guides were made available alongside the data capture graphs. The second UI was accessible only to site coordinators and principal investigators (Fig. 3), which allowed them to view the RATE score for all participants assigned to a subgroup, along with the trends over time. Principal investigators can sort participants by RATE score and select an individual participant to visualize the participant’s RATE Tracker summary as well as interact with survey results for the participant.

#### Offline research environment

The study platform also has an offline environment that allows researchers and data scientists to efficiently explore and develop with any new incoming data in a Python framework (Supplementary Fig. 2). This research environment has a clone of the deployed system, with survey data annotations and wearable data being synced daily for machine learning training. Researchers and data scientists were able to take advantage of this environment to: 1) perform exploratory feature engineering, 2) explore several data quality and missingness metrics, 3) perform automatic labeling for model training or updating, and 4) test next generation machine learning models and compare them with the current deployment.

The research and development environment also enables reproducible research, in that machine learning dataset can be efficiently prepared, versioned and shared with researchers through a functional application interface (API). For example, in the functional API subjects can be divided in batches based on user id, in a way that is uniform both in terms of data collection time and COVID status (which is automatically updated based on any new input survey data). Access to the dataset labels was only allowed via this functional API, in order to ensure proper comparisons and minimize label leaks as the team of developer worked in parallel during feature and model engineering tasks.

### Predictive model training applied to COVID-19

#### Cohort selection and machine learning dataset

**Figure 4:**
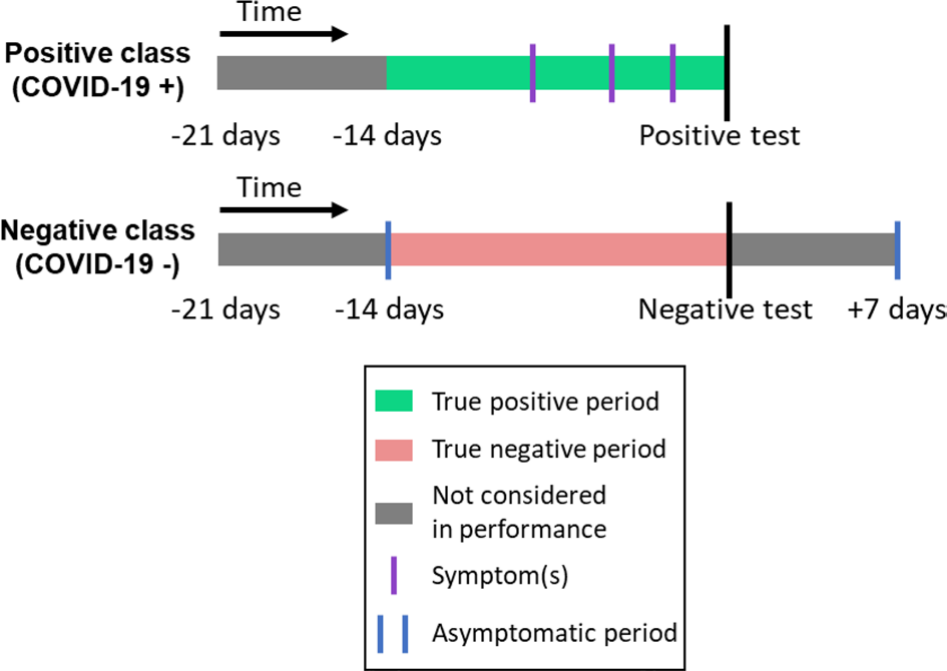
Graphical summary of definitions for positive and negative classes.

We used COVID-19 test results as the primary criterion to define our cohort for binary classification (Fig. 4): the positive class was defined to be subjects who reported a positive test result, and the negative class was defined to be subjects who reported at least 1 negative test result, but no positive result. In the case of multiple negative test results for subjects in the negative class, the test date with the most collected physiological data was chosen as the fiducial point for model training and validation.

In addition, we required subjects in both classes to have sufficient data, defined by the following three creteria:

1. subjects must have had ≥ 10 nights of physiologic data collected during sleep within the 21-day period prior to their COVID-19 test;
2. subjects in the positive class must have reported symptoms within the 14-day period prior to their positive COVID-19 test, while subjects in the negative class must have not reported any symptoms within the 14 days prior to and the 7 days following their negative test (Fig. 4, asymptomatic period);
3. subjects must have had physiologic data collected simultaneously from both the Garmin watch and Oura ring.

For model training, study subjects were split into five stratified folds with the 5th fold being a vaulted test set. These folds were made consistent across the data collection process and enforced for all users of the system via a functional API in order to minimize label leaks. The model was trained on the first four folds using four-fold cross-validation. To assess the performance of the algorithm, we defined a true positive as being a positive model prediction within the 14-day period prior to a positive COVID test for the positive class (Fig. 4, True positive period), and a true negative as being a negative model prediction within the 14-day period prior to a negative COVID test for the negative class (Fig. 4, True negative period). Study principal investigators suggested reporting performance at a threshold of 60% sensitivity. Thresholds achieving this sensitivity from the cross-validation folds were averaged to yield a single threshold, which was tested on the vaulted 5th fold. The reported results were from the four-fold cross validation in addition to the 5th fold held out test.

#### Feature extraction

To minimize the impact of everyday activities and other contextual factors on physiology, the physiological data were extracted from the raw wearables data only during sleep periods. Plausibility filters were applied so that unrealistic measured values outside of a very broad physiological range were discarded ( see Supplementary Table 5 for a detailed list of valid ranges per physiological measurement). This action resulted in a segmented time-series for each physiological measurement type (e.g., heart rate or temperature), with each segment corresponding to a distinct sleep period (see Supplementary Fig. 1 for an example).

The core features extracted from the sleep period physiological data can be categorized into three groups: (1) sleep period statistics; (2) trend statistics; and (3) deviations from personal baseline. All three categories of features attempt to capture abnormal physiological attributes, albeit in different ways. Statistics on the sleep period features captured distributional information of the physiological data from the last available sleep period, including derived features like maximum, minimum, median, mean, standard deviation and inter-quartile range.

The trend statistics and deviations from personal baseline features attempted to capture distributional shifts of the physiological data over multiple sleep periods. The trend statistics used a variation of the Mann–Kendall test^33^ to robustly estimate monotonic increasing and decreasing trends. For a given physiological input (e.g., temperature), let $x_{1} ,x_{2} , \ldots ,x_{N}$ denote the sleep period segmented time-series over the past N nights, with $x_{i}$ a vector of measurements collected during the ith sleep period. The estimated monotonic trend over the past k sleep periods, is given by the following:

$${\text{trend}}_{k} = \frac{1}{m}\sum\limits_{i = 1}^{k + 1} {\sum\limits_{j = i + 1}^{k + 1} {\sum\limits_{p} {\sum\limits_{q} {{\text{sign(x}}_{ip} - x_{jq} )} } } }$$

where p and q range over the measurements in each sleep period and m is the total number of sign comparisons made in the above calculation. The above trend statistic is bound to the range [− 1, 1], with − 1 indicating a perfectly monotonic decreasing trend, + 1 indicating a perfectly monotonic increasing trend, and 0 indicating no trend at all. For feature extraction purposes, we calculate the above for k = 1,2,…,7.

The deviations from personal baseline features were estimated from the distribution of z-score values of the respective feature observed over an observation window. The feature value was converted to z-score by comparing with the distribution of the corresponding feature for each participant in a baseline window, which is a 10-day period with a gap of 7 days from the present time, on a rolling basis. We required the 10-day baseline period to have a group of seven consecutive days in which the following two criteria were met:

- There were at least 5 good sleep periods over days;
- A good sleep period occurred, as characterized by a minimum of three hours of data collection.

Once the baseline window was established, we computed mean and standard deviation for every feature of interest over this period to be used in the z-score calculations. We then used mean and standard deviation from the distribution of z-score values of the respective feature in the observation window as input to the classification model.

#### Modeling approach

**Figure 5:**
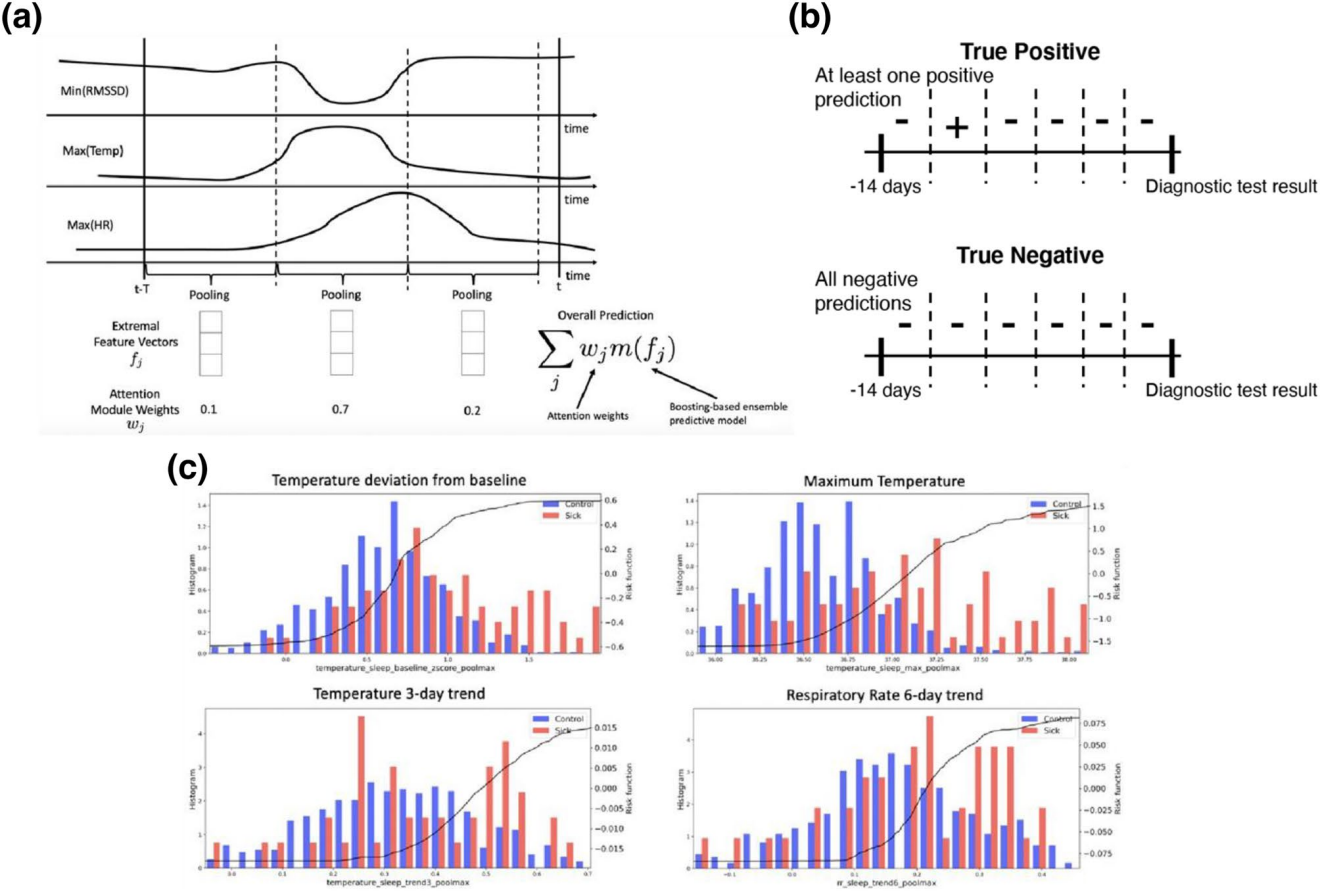
(**a**) Illustration of sliding window-based feature extraction. (**b**) Example of true positive and true negative classification under multiple instance learning training objective. (**c**) Illustration of learned feature risk scores (black curves) along with population distribution underlays for Sick (red) and Control (blue) populations included in the infection prediction mode.

A multi-resolution sliding window-based technique was used to extract a set of extremal feature vectors to capture abnormal physiological features over a given time window by using max- or min-pooling. An attention module was employed to localize the most discriminative time window within the 14-day observation period prior to COVID diagnostic test for each example. This was achieved by learning a weighted model prediction over sliding windows within the 14-day period that concentrated the weights on the best performing sliding window using a SoftMax function^34^ (see Fig. 5a). This was combined with a training objective adapted from multiple instance learning that treats sliding windows from a given example as a collective unit. For COVID-19 positive cases, the training objective considered a true positive prediction for the given sample if at least one of the sliding windows predicted COVID positivity. For COVID-negative cases, the training objective considered a true negative prediction if all of the sliding windows predicted COVID-negative status – see Fig. 5b for an example.

The underlying predictive model utilized by the technique was based on a custom gradient boosting ensemble learning method coded in Python that enabled the model predictions to be interpretable. For each physiological feature, a directionality constraint was imposed on its utilization that forced the estimated model prediction to be monotonically increasing or decreasing with respect to that feature. For example, it is well known that temperature is expected to rise in response to an infection; as a result, the model was constrained so that its output was monotonically increasing functions of features based on temperature (including statistics, trends, and deviations from baseline). Similar constraints were applied to the other physiological inputs (increasing constraint for respiration rate and heart rate features, decreasing constraint for heart rate variability features). Figure 5c illustrates a number of features included in the model; notice that the black risk curves in each plot exhibit a monotonic pattern.

## Results

### System prototype performance applied to COVID-19 infection

**Figure 6:**
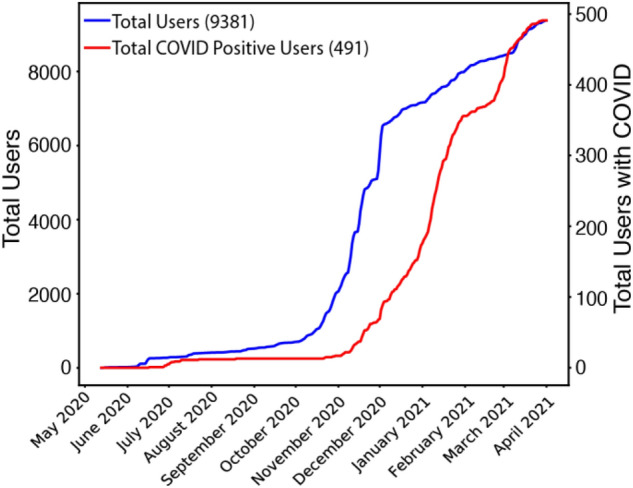
Timeline of total users and COVID + users through April 1, 2021.

We deployed the first system prototype on June 23, 2020, and over the next 10 months, continuously enrolled participants and maintained the system for collection and processing of wearables data. At the conclusion of the study in early April 2021, a total of 9381 users had enrolled, with 7458 (79.5%) males, 1922 (20.5%) females, and 1 other/unknown; see Table 1 for age demographics. Overall, 491 participants reported a COVID-19 diagnosis sometime during their participation in the study. A graph of the total number of study participants and COVID-19 positive subjects over time is shown in Fig. 6. The total number of participants (blue line) increased considerably during the period of early October 2020 to late November 2020. Correspondingly, the total number of COVID-19 positive subjects (red line) sharply increased during the period of early November 2020 to late January 2021. In total, our system collected 201 million hours of wearables data and delivered 599,174 total user-days of predictive service (median of 62 days of service per participant).

**Table 1:** Age demographics of the study participants.

Age category	Participant count (%)
18–34 years old	5865 (62.5%)
35–49 years old	2804 (29.9%)
50–64 years old	680 (7.2%)
65–74 years old	30 (0.3%)
75–84 years old	0 (0%)
> 84 years old	2 (< 0.1%)

Over the 10-month study period, our system was operational over 99% of the time. System downtime was primarily due to 2 major system upgrades that we deployed in December 2020 and April 2021 to help maintain system stability with the increasing number of study participants. We also released periodic updates to the user UI and user daily surveys that resulted in brief system downtimes. Additionally, some updates experienced unforeseen run-time problems that were caught by study principal investigators and that had to be quickly diagnosed and repaired. Other reasons for lapses in predictive service that were not related to system upgrades included delays in data synchronization from the COTS device vendors to our system.

### Predictive algorithm performance applied to COVID-19 infection

**Figure 7:**
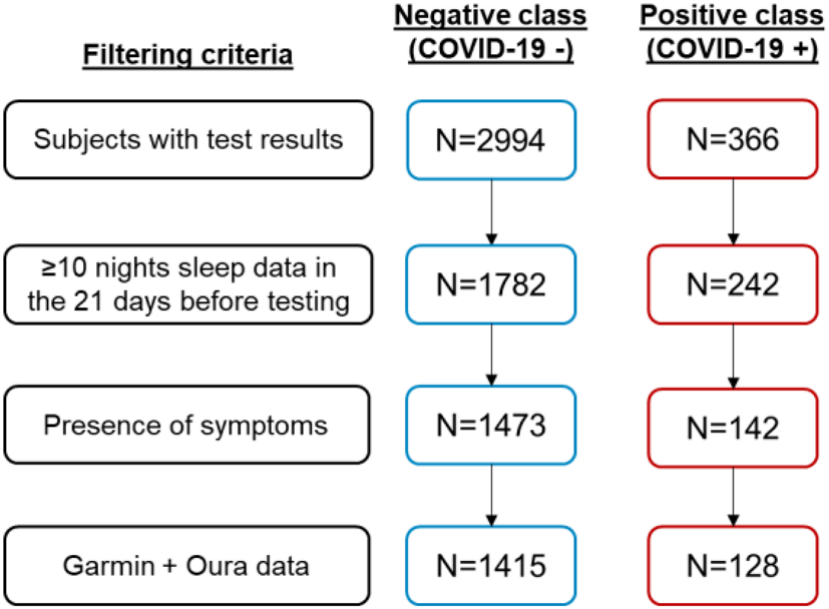
Positive and negative class size in the machine learning dataset at each filtering step.

While there were a total of 491 COVID + subjects at the conclusion of the study, we used only a subset of them for model training and validation. We constructed a machine learning dataset from subjects enrolled in the study up to February 18, 2021 (approximately 1.5 months prior to the conclusion of the study). There were 2994 subjects who fit the primary COVID-19 testing criterion for the negative class, and 366 subjects who fit the criterion for the positive class, as shown in Fig. 7. When filtering by adequate physiological data during sleep, the cohort size reduced to 1782 and 242 for negative and positive class, respectively. After excluding asymptomatic COVID positive subjects and COVID negative subjects who reported symptoms around the time of their COVID test, there were 1473 subjects in the negative class and 142 subjects in the positive class. Finally, we filtered for subjects who had worn both the Garmin watch and Oura ring simultaneously, which resulted in 1415 and 128 in the negative and positive classes, respectively, and these subjects were used for model training and validation.

**Figure 8:**
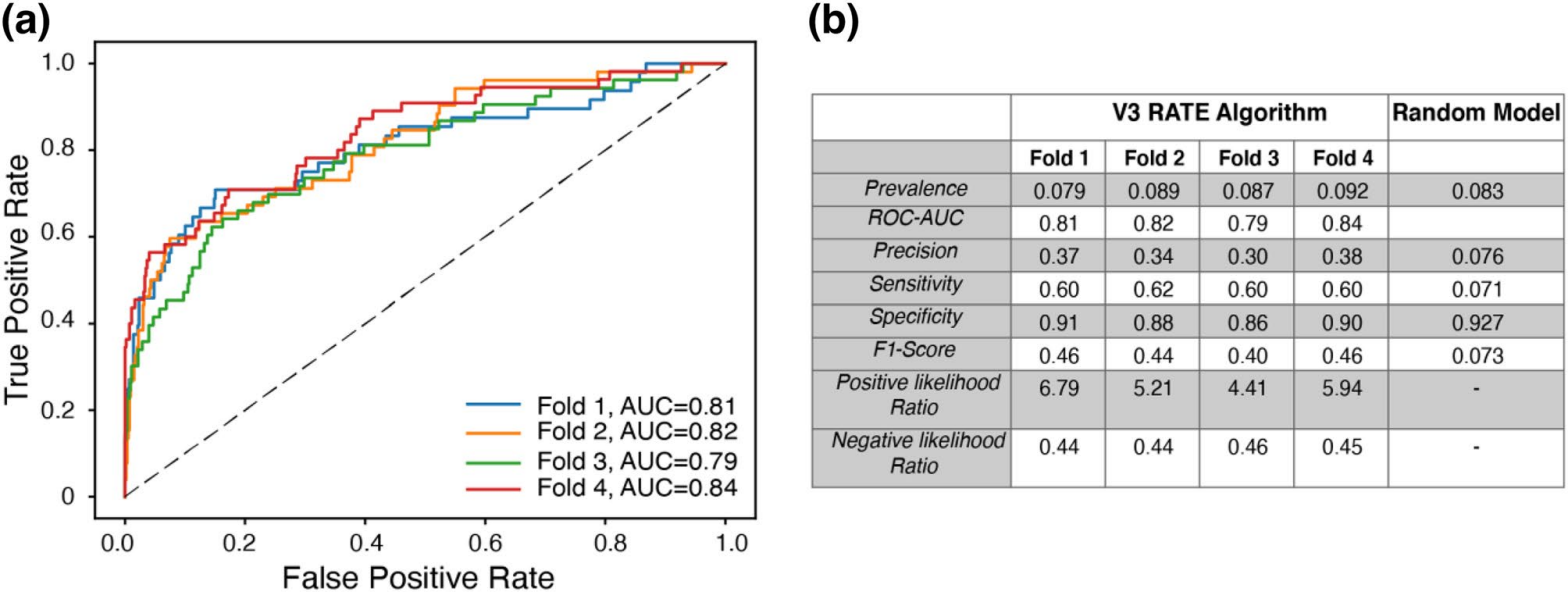
(**a**) ROC curve of the algorithm for each fold. Dash line represents guessing. AUC is indicated for each fold in the legend. (**b**) Infection predictive model performance. Random model results based on dummy classifier using stratified approach with random guessing proportional to the class distribution/prevalence (~ 9%).

The RATE algorithm demonstrated a viable performance with a mean cross-validated ROC-AUC of 0.82 ± 0.02 (Fig. 8a). As shown in Fig. 8b, the precision of the model is 4.4 × better and F1 score 6.3 × better than a random approach (in which COVID-19 positive predictions are made randomly in proportion to the class prevalence). The RATE predictive score was presented on a normalized scale in the range of 0 to 100, with larger scores indicating a higher likelihood of infection. A sensitivity of 60% corresponded to a RATE score threshold of 11, which is more than 5 times greater than the population baseline score of 2. Note, reported performance is for the field-deployed prototype, not a hypothetical exploration, assuming imaginary implementation or ideal data conditions.

In addition to assessing the selected RATE score threshold of 11 on the held-out test set, we also assessed it on COVID-19 positive cases that were collected after the February 18 cutoff. Between February 18 and April 1 (the conclusion of the study), an additional 51 subjects reported a COVID-19 positive diagnosis. Of these 51 subjects, 29 exhibited a RATE score threshold crossing above 11, which closely mirrored the 60% sensitivity target.

**Figure 9:**
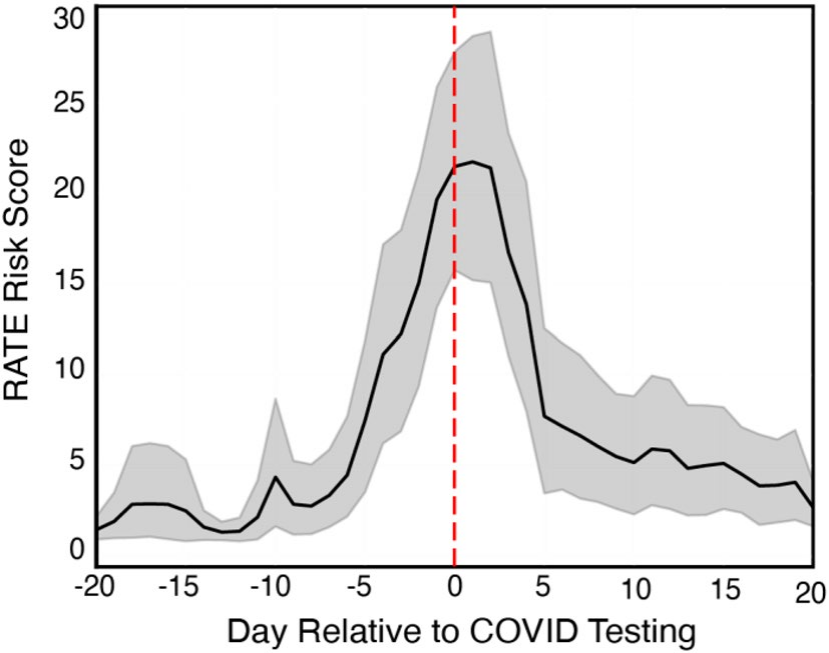
Mean RATE risk score in 179 COVID-19 positive + users as a function of day relative to COVID testing (red line). Grey region depicts 95% confidence interval (standard error).

We also examined the temporal patterns of the RATE score on the 128 COVID + subjects in the machine learning dataset and the 51 cases collected afterwards (179 total COVID +). For these 179 subjects, we found that the RATE score began to rise as early as 6 days prior to COVID testing, with the highest risk scores around the time of testing and up to 2 days following the test, as shown in Fig. 9. To estimate the overall lead time of positive classification, we identified the days in which the RATE score exceeded the defined threshold within the 14-day window prior to COVID testing. The lead time was then defined as the average across these positive days for each user and then aggregated across the cohort for the final mean estimate of the lead time for COVID classification. Based on a cut-off risk threshold of 11 (yielding 60% sensitivity), the RATE algorithm successfully predicted COVID, on average, 2.3 days prior to testing. Note that this lead time neglects data arrival latencies that were experienced by the study platform deployment (see Discussion), which effectively eroded some of the early warning power.

## Discussion

Maintaining workforce readiness and productivity during a pandemic is challenging but critical for essential workers. To address this challenge, US DoD has invested in exploring a novel approach, where each member of a workforce serves as their own "early warning sentinel" with automation continually monitoring their changing health status. A unique aspect of this study—to arrive at a prototype of the sentinel—was the consistent sense of urgency, fostered by the COVID-19 pandemic, to seize the opportunity for data collection, prospective testing and validation, and iterative development of the study platform. The tight collaboration (typically daily) between the research team and the DoD informed a series of rapid incremental improvements to the prototype system, evolution of the web-based UI, and new predictive machine learning models. Results showed fast progress in: system maturity; real-time service delivery; usability; and design of usage workflow within operational deployments (the objectives of the project), and also utility of the predictions.

This study was able to:

- Design and develop a scalable and operational system that delivers an early warning real-time service for prediction of infection;
- Evaluate the feasibility and performance of the system (technical, usability, and utility) while it was being used for operational work (as opposed to solely an academic retrospective assessment of its potential under ideal laboratory conditions).

We demonstrated feasibility. The algorithm predicted symptomatic SARS-CoV-2 from COTS wearables data alone with an AUC of 0.82 on average 2.3 days prior to COVID test amongst participants with at least 48% wearable data available during sleep periods. At an operating point of 60% sensitivity, the false positive rate was observed to be 11%, which is evaluated over a 14-day period. When considered as a daily testing rate, this amounts to < 1% daily testing rate. While these results are promising and show the potential for monitoring workforce readiness, the following highlights some of the remaining technical challenges that limit operationalization of this technology at present and inform future work.

A crucial factor for success is controlling for and managing data fidelity issues arising from noise and data missingness due to confounding factors, user compliance, and overall system deficiencies (issues relating to wearable device configuration and/or cloud integration). This study tackled this challenge from two perspectives. First, UI design sought to maximize wearables compliance via guided training materials, visualizations of device capture and data quality, and tooltips and troubleshooting guides. User fatigue in completing the daily survey prompted modifications to the UI that allowed participants to retrospectively submit multiple surveys (e.g., on a weekly basis). Second, algorithmic tools were deployed to mitigate the impact of data quality on the prediction. These included leveraging the available activity and hypnogram data to extract physiological data during resting periods and using ensembles of predictive models for adapting to missing features. As the use of activity data was found to be very important in resolving a clean physiological signal, better accounting for this meta-information is an ongoing and active area of work. In addition, although a viable predictive performance was achieved from 48% wearable data available (as calculated based on the number of sleep periods with physiological data), a better characterization of the tradeoff in data availability versus predictive performance is an area of future work.

Another data integration factor that was identified over the course of the study was latencies in data arrival to the study platform (observed to sometimes be as large as 24–48 h). This directly impacted the timeliness of the predictive score and eroded some of the predictive lead time of the algorithm. Upon further investigation, this proved to be a complex issue with multiple causes: frequency of device syncing by participants, data processing and queueing by COTS vendors, and transfer and processing by the study platform. Although the latency issues have been improved with enhancements to software and infrastructure, better standards and protocols, possibly through a consortium of commercial parties, may also streamline and accelerate this progress. At present, the study platform has limited control and awareness of software and firmware updates made on the wearable devices, which may negatively impact the data quality, availability, and integration affecting the overall functioning of the platform.

Several learnings were derived from the training and validation of the infection prediction models. First, the supervised labels for training the predictive models were derived from self-reported daily surveys, which may introduce errors in the ground-truth labels. To mitigate this effect, fiducial points were derived from the administration of COVID-19 tests, which was perceived to be more objective and specific than self-reported symptoms. Second, participant feedback recommended enhancing the UI with visualizations to explain the causes of an infection prediction score made by the model; this will be pursued in future work. This point drove the model training to focus on interpretable predictive models over deep learning techniques. Field testing showed that graphs of a participant’s infection risk score over 1-, 2-, and 3-week periods proved to be useful in allowing principal investigators to properly baseline a participant’s current score. Our testing corroborates findings from this study and others in the literature^25–31,35^, that suggest that relative changes in physiological signals as compared to an earlier baseline estimate are very important in capturing the predictive signal. Finally, the logic that is applied to detect a COVID-19 case (e.g., selecting a threshold on the infection risk score) involves a complicated trade-off between sensitivity, specificity, and the predictive lead time of the algorithm. As the threshold is increased, one expects specificity to increase at the expense of decreased sensitivity and decreased predictive lead time. Better visualizations of this trade-off are required to calibrate the algorithm to the requirements of the intended use case.

The algorithm development proceeded iteratively as more wearables data became available via the study. Although model improvements were developed and validated in an offline fashion, future work may explore training methodologies that adjust the model in an online fashion as data is streamed to the platform;^36,37^ or an anomaly detection to identify significant deviations from training cohort and/or mitigate effects of COTS firmare updates and service downtimes. The initial predictive model, trained in the early stages of the study when few COVID + cases had been acquired, was bootstrapped from a Philips proprietary dataset of hospitalized patients^32^. Although the considered physiological inputs in the hospital data were constrained to match those acquired via wearables, a number of significant differences limited the translation of the model to this study. Firstly, a significant covariate shift was identified between the skin temperature measurements acquired from the Oura ring and the temperature measurements acquired in the hospital setting (core body temperature). Secondly, some features like oxygen saturation that were found to be predictive in hospitalized patients showed limited predictive power in the wearables study, which was due to quality degradation between a medical device and a COTS wearable, and potentially pathophysiology differences between the participants in the wearables study and the hospitalized patients. Future work will further explore the source of differences and methodologies to transfer models between these two domains.

A summative questionnaire was administered to 24 principal investigators (not individual participants) near the end of the project to illuminate user preference and utility of the system. Subjective results regarding future usage of the RATE predictive service are summarized in Supplemental Material Fig. 3a-f. Large individual differences in responses highlight broad future applicability and the need for a flexible application approach.

In December 2020, nine months into our data collection study, the Food and Drug Administration (FDA) issued an emergency use authorization (EUA) of COVID-19 vaccines. Although the daily survey questionnaire was modified shortly thereafter to include questions about dates of vaccine doses received, these data have not yet been fully analyzed at the time of writing this article. Preliminary analysis suggests that on average, the cohort infection score increases on the night of the second vaccination, peaks between 1–3 days after, and returns to baseline levels by the fifth day. However, fully characterizing the similarities and differences between the physiological patterns of COVID-19 and vaccine administration is future work.

## Supplementary Information

Supplementary Information.
