# Supplementary material for: Real-time infection prediction with wearable physiological monitoring and AI to aid military workforce readiness during COVID-19
Source: Sci Rep. 2022 Mar 8;12:3797. doi: 10.1038/s41598-022-07764-6 (PMC8904796; doi:10.1038/s41598-022-07764-6)
Supplement: Supplementary file 1 — Supplementary Information. [file 41598_2022_7764_MOESM1_ESM.doc]

Real-time infection prediction with wearable physiological monitoring and AI to aid military workforce readiness during COVID-19

Bryan Conroy^1^, Ikaro Silva^1^, Golbarg Mehraei^1^, Robert Damiano^1^, Brian Gross^1^, Emmanuele Salvati^1^, Ting Feng^1^, Jeffrey Schneider^2^, Niels Olson^2^, Anne G. Rizzo^3^, Catherine M. Curtin^4^, Joseph Frassica^1,5^, Daniel C. McFarlane^1*^

^1^ Philips Research North America, Cambridge, MA, USA

^2^ Defense Innovation Unit, Mountain View, CA, USA

^3^ The Guthrie Clinic, Sayre, PA, USA

^4^ Department of Surgery, Palo Alto Veteran Affairs Healthcare System, Palo Alto, California, USA

^5^ Institute for Medical Engineering and Science, Massachusetts Institute of Technology, Cambridge, MA, USA

* Corresponding author email address: dan.mcfarlane@philips.com

# Supplemental Material

Supplementary Table 1: Comparison of some methods for containing pandemic

| **Approach** | **Pros** | **Cons** |
| --- | --- | --- |
| Vaccine | Immunized resistance | Slow to develop; limited to a specific infectious agent; mutations of the target pathogen can defeat a vaccine; possible side-effects^38^ |
| Directly sense the presence of infectious agent (e.g., surfaces, in the air, in the water, etc.) | Early warning of threat | Requires custom development; limited to a specific infectious agent; limited range; collecting samples to test can be labor-intensive^39^ |
| Contact tracing regarding proximity to positively-diagnosed individuals | Warning of exposure | Privacy issues; does not warn of contact with infected and contagious people who are not yet diagnosed; compliance problems^40^ |
| Symptom tracking | Pre-diagnosis warning of risk^41^ | Before symptoms appear, people may have already been contagious and spreading infection to others for multiple days^42^ |
| Diagnostic testing of an individual sample | Diagnose infection in an individual^43^ | Tests are narrowly specific to particular pathogens, and must be developed custom for each new type of infectious agent; there is a delay in development and deployment of tests; mutations of the pathogen can defeat the test; producing test results can take significant time; can be difficult and costly to administer; and must be frequently repeated^44^ |
| Diagnostic testing of pooled samples from multiple individuals | More economical than individual testing; a negative result can clear an entire group | (Similar 'Cons' as testing for individuals) if results are positive, the entire group must additionally be individually tested (so the delay for receiving results is doubled)^45^ |
| Work remotely; work from home; general isolation | Prevent exposure | Multiple essential types of work cannot be done remotely; degraded productivity for many other types of work; social isolation negatively affects mental health^46^ |
| Screening for key indicators of infection: presence of symptoms, travel history, spot-check temperature | Screening of symptomatic or other high-risk individuals^25^ | Does not identify people who are pre-symptomatic or asymptomatic; subjective; vulnerable to deception; can create crowded choke points that increase the risk of transmission^47^ |
| Personal protective equipment | Prevent the physical spread of pathogens | Widespread compliance is extremely difficult, especially for: individuals without symptoms, children, and disabled adults; and it is uncomfortable and interferes with normal activities like eating and social interaction^48^ |
| Physical barriers at key points of human-human interaction in the workplace | Prevent the physical spread of pathogens | Many types of human-human interaction take place in locations that are impractical to protect with barriers; partial barriers only offer partial protection^49^ |
| Hand sanitization methods | Removes pathogens from hands | Requires frequent repetition; no protection from airborne threats; no protection between sanitization events; inconvenient; widespread compliance is very difficult^50^ |
| Surface sanitization processes | Removes pathogens from surfaces | Infeasible on some surfaces; requires frequent repetition; can be labor-intensive; no protection from airborne threats; large-scale deployment is difficult^51^ |
| Social distancing | Reduces contact | Limited effectiveness; compliance is very difficult; does not prevent the deposition of pathogens in the environment^52^ |
| Context-based predictions – nexus | An estimation of general risk | Requires accurate information about the context; no identification of infected individuals; limited scope^53^ |
| Support for context | Decrease general risk by improving the context | Impractical for some contexts; difficult to maintain context change^54^ |
| **Physiological monitoring with wearables and real-time machine learning to infer pre-symptomatic infection (the topic of this article)** | **Early warning of threat;** **potential early warning of unknown pathogens; effective with mobile workers** | **Compliance is somewhat challenging; dependent on the quality of the wearable device(s) deployed; Requires data for training/tuning a machine learning model** |

Supplementary Table 2: Summary of physiological measurements captured by the COTS devices

|  | **Garmin Fenix 6** | **Garmin Vivoactive 4** | **Empatica E4** | **Oura Ring** |
| --- | --- | --- | --- | --- |
| **Monitoring Paradigm** | 24/7 | 24/7 | 24/7 | Skin temp 24/7, IBI sleep time only |
| **Heart Rate** | Every 15 sec | Every 15 sec | 10-second average updated every 1 sec | Derived from IBI |
| **PPG** | Does not provide raw signal | Does not provide raw signal | Raw PPG sampled at 64 Hz | Does not provide raw signal |
| **Inter-Beat Interval** | - | - | 1/64 sec resolution | 1 millisecond resolution |
| **Respiration Rate** | Updates every minute | Updates every minute | Can be derived from PPG | Nighttime average (one value per night) |
| **Pulse Oxygenation** | Updates every 5 minutes | Updates every minute | - | - |
| **Temperature** | - | - | Skin temp. sampled at 4 Hz | Skin temp. updated every minute |
| **Acceleration** | Does not provide raw signal | Does not provide raw signal | Raw ACC sampled at 32Hz | Does not provide raw signal |
| **Additional Sensors** | - | - | Galvanic Skin Response (GSR) | - |
| **Derived Features** | Motion intensity, VO2, Sleep report, Stress, Calories expenditure | Motion intensity, VO2, Sleep report, Stress, Calories expenditure | RR, Heart rate variability (HRV) | Coarse grained activity, Sleep report, HRV |

Supplementary Table 3: Device integration method with COTS wearables

| COTS Vendor | Integration Method |
| --- | --- |
| Garmin | Integration with Garmin watches was achieved by creating a Garmin Connect Developer account that enabled Garmin watch participants to enroll in the data sharing study via an OAuth authentication process. Once authenticated, an access token linked to that participant was shared with the study platform. Data sharing was then enabled via a “push” mechanism, in which the Garmin cloud pushes data to the study platform when participants synchronized their watches via the native Garmin mobile application. A RESTful API over HTTPS was deployed on the study platform to receive these data pushes from Garmin and generate a cached version of the standardized and compressed data format ( as described in Supplementary Table 5 ). |
| Oura | Integration with Oura rings was achieved by creating an Oura Teams account that enabled Oura ring participants to consent to share their data with the study via a secure authentication step during registration. Due to the lack of a push mechanism available at the time of the initial deployment, participant data were automatically downloaded from the Oura cloud without the collection of any personally identifiable information via custom software that was scheduled to run frequently on the study platform. A 24-48-hour delay in data collection via this method was observed that impacted the overall predictive lead time of the infection risk score. The Oura engineering team collaborated to develop a "push" mechanism for data sharing that vastly improves the observed delays in data availability. As of the time of this writing, this new mechanism is being integrated into the research prototype platform. |

Supplementary Table 4: Daily survey questions. Note the responses outlined below – ‘>’ indicates ‘button’,‘●’ indicates radio button (single response only),‘□’ indicates checkbox (multiple options available).

| Questions | Response options |
| --- | --- |
| Have you been tested for infection since the last time you completed this survey? (Regardless of whether the results are available yet.) | No new testing  ● Yes, I was tested for...  □ COVID-19, SARS-CoV-2  ● No symptoms  ● Feeling ill  □ Flu, influenza  ● No symptoms  ● Feeling ill  □ Strep throat  ● No symptoms  ● Feeling ill  □ Other bacterial  ● No symptoms  ● Feeling ill  □ Other viral  ● No symptoms  ● Feeling ill  □ Other fungal  ● No symptoms  ● Feeling ill  □ Other don't know  ● No symptoms  ● Feeling ill |
| When did you receive this test(s)? | ● Last 1-2 hours  ● Last 12 hours ● Last 24 hours  ● More than 24 hours  ● I’m not sure |
| Have you received results of a test(s) for infection that you have not yet reported here? | No new results >  ● Yes, I received results of testing for...  □ COVID-19 (SARS-CoV-2) test results  ● Positive – no symptoms  ● Positive – feeling ill  ● Negative  □ Flu (influenza) test results  ● Positive – no symptoms  ● Positive – feeling ill  ● Negative  □ Strep throat test results  ● Positive – no symptoms  ● Positive – feeling ill  ● Negative  □ Other bacterial test results  ● Positive – no symptoms  ● Positive – feeling ill  ● Negative  □ Other viral test results  ● Positive – no symptoms  ● Positive – feeling ill  ● Negative  □ Other fungal test results  ● Positive – no symptoms  ● Positive – feeling ill  ● Negative  □ Other don't know test results  ● Positive – no symptoms  ● Positive – feeling ill  ● Negative |
| When did you receive the result(s)? | ● Last 1-2 hours  ● Last 12 hours ● Last 24 hours  ● More than 24 hours  ● I’m not sure |
| Have you felt any symptoms since the last time you completed this survey? | No new symptoms >  ● Yes, I have had symptoms...  □ Fever  ● Under 99.1 F (37.3 C)  ● 99.1-100.4 F (37.3-38.0C)  ● 100.5-102.2 F (38.1-39.0 C)  ● Over 102.2 F (39.0 C)  □ Cough  □ Shortness of breath or difficulty breathing  □ Chills  □ Repeated shaking with chills  □ Muscle pain  □ Headache  □ Sore throat  □ New loss of taste of smell  □ Fatigue  □ Sputum, congestion, runny nose  □ Other |
| When did you first start feeling these symptoms? | ● Last 1-2 hours  ● Last 12 hours ● Last 24 hours  ● More than 24 hours  ● I’m not sure |
| Have you taken any medications or received any vaccinations since you reported last? | ● Yes, new medications or vaccinations...  □ COVID Vaccine   - Moderna (1^st^ of 2) - Moderna (2^nd^ of 2) - Pfizer (1^st^ of 2) - Pfizer (2^nd^ of 2) - Other single dose vaccine - Other 2 dose vaccine (1^st^ of 2) - Other 2 dose vaccine (2^nd^ of 2)   □ Advil®, Motrin® (ibuprofen)  □ Tylenol® (acetaminophen)  □ Aspirin (acetylsalicylic acid)  □ DayQuil®, NyQuil® (Cold & Flu)  □Benadryl®(antihistamine, diphenhydramine)  □ Sudafed® (pseudoephedrine)  □ Antibiotics  □ Other medication  □ Flu shot, influenza vaccination  □ Other vaccination |
| When did you take these medications or receive these vaccinations? | ● Last 1-2 hours  ● Last 12 hours ● Last 24 hours  ● More than 24 hours  ● I’m not sure |

Supplementary Table 5: Summary of physiological upper and lower bounds ( inclusive) used by the plausibility filter.

| **Physiological Measurement** | **Lower Bound** | **Upper Bound** |
| --- | --- | --- |
| Skin Temperature ( Celsius ) | 28 | 41 |
| Heart Rate ( beats per minute ) | 30 | 220 |
| Respiratory Rate ( breaths per minute ) | 4 | 30 |
| SpO2 ( percent ) | 80 | 100 |

Supplementary Table 6: Summary of standardization format used to cache device data into Pandas data frame for further use the by system. The “tm” column was also used as the data frame index.

| **Physiological Measurement ( units )** | **Column Name** | **Data Type** |
| --- | --- | --- |
| Data collection time ( Unix epoch time, in seconds ) | tm | uint32 |
| Heart Rate ( beats per minute ) | hr | float64 |
| Respiratory Rate ( breaths per minute ) | rr | float64 |
| SpO2 ( percent ) | spo2 | float64 |
| Inter Beat Interval (IBI) ( milliseconds) | ibi | float64 |
| IBI Root Mean Square of successive differences ( milliseconds ) | rmssd | float64 |
| Skin Temperature ( Celsius) | temperature | float64 |
| Hypnogram ( categorical ) | hypnogram | categorical |
| Motion ( categorical ) | motion | categorical |

**
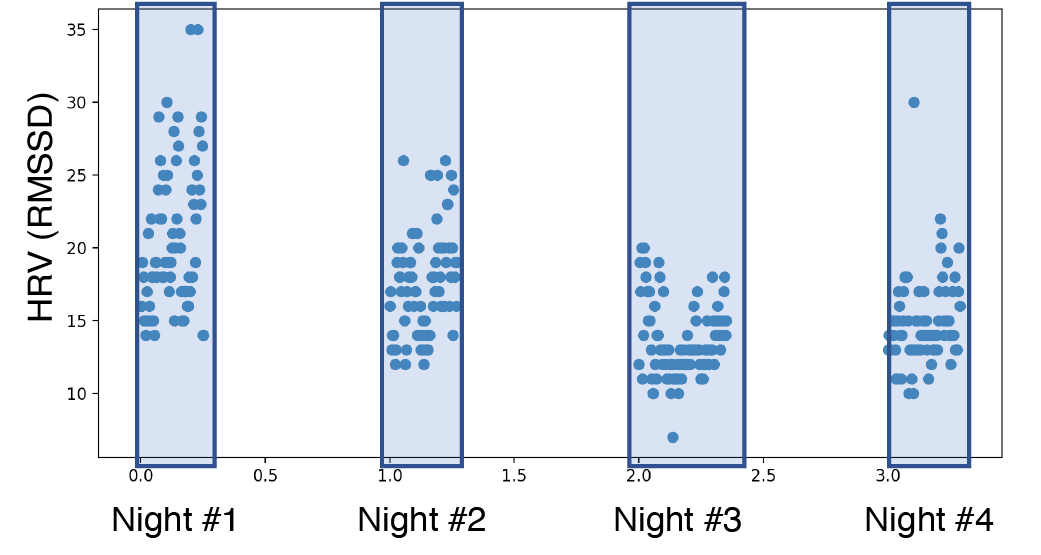
**

Supplementary Figure 1: Illustration of heart-rate variability (RMSSD) data after sleep period segmentation.


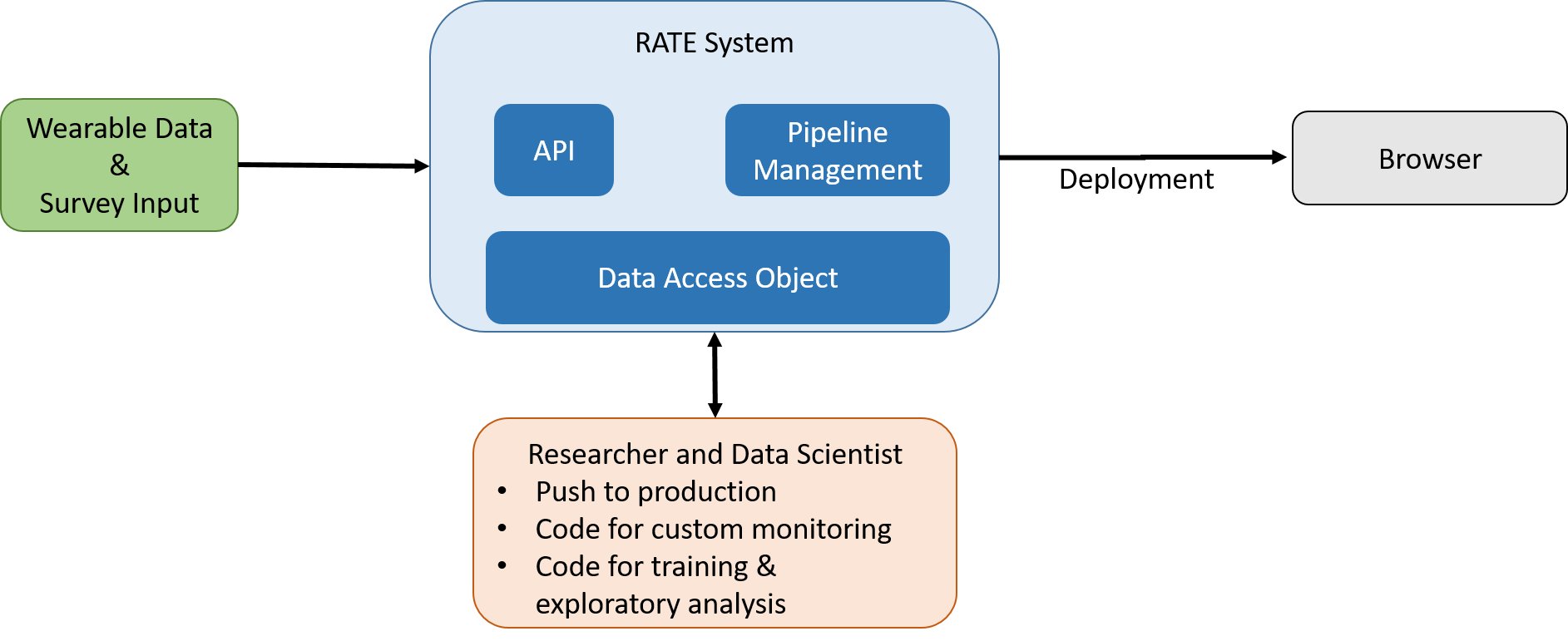


Supplementary Figure 2: The study platform was designed to facilitate efficient development and deployment of new machine learning models into the runtime execution environment.


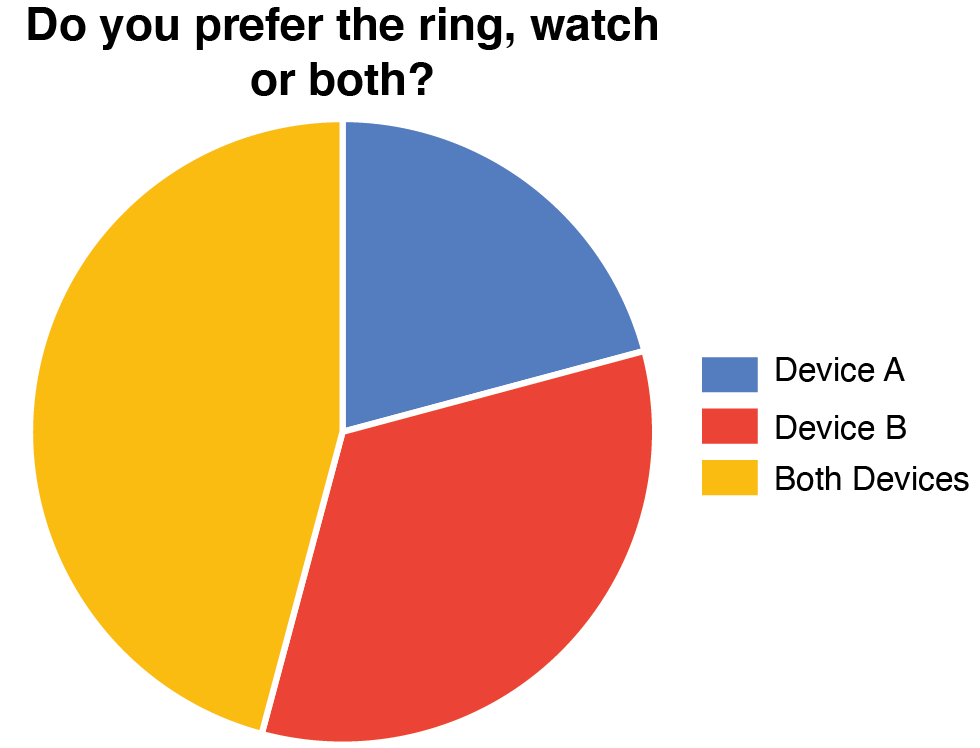
Supplementary Figure 3a: Survey results (Most responses were collected in March 2021). Principal investigators (24) commented about their preference for selection of wearable(s) for use with the RATE service. The actual survey question was, "Do you prefer the ring, watch or both?" Responses of "watch" or "ring" are commercially-sensitive and coded here without a key as "Device A" and "Device B." Results highlight a strong individual differences effect with no clear one-size-fits-all wearable solution. About 46% responded that they prefer both, and the remaining 54% disagree over which single wearable they favor.


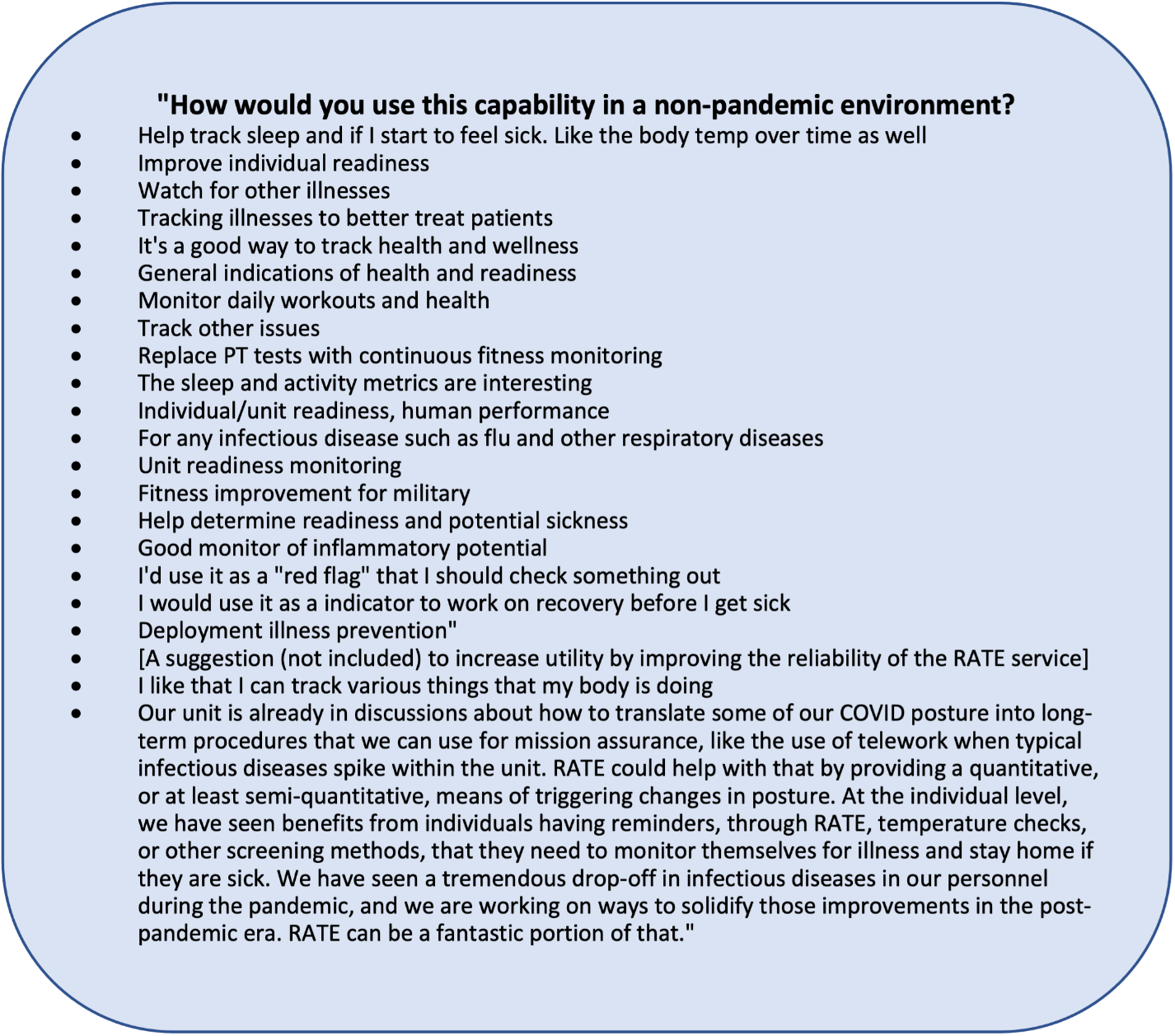
Supplementary Figure 3b: Survey results (Most responses were collected in March 2021). Principal investigators (24) were asked to share their ideas for future use of RATE after the end of the current COVID-19 pandemic. The actual survey question was,, "How would you use this capability in a non-pandemic environment?" Results highlight the broad potential utility for RATE in supporting maintenance of workforce readiness. Ideas for use include general monitoring, tracking, and early warning for physiological changes related to: infection, physical fitness, activity, wellness, health, and illness.


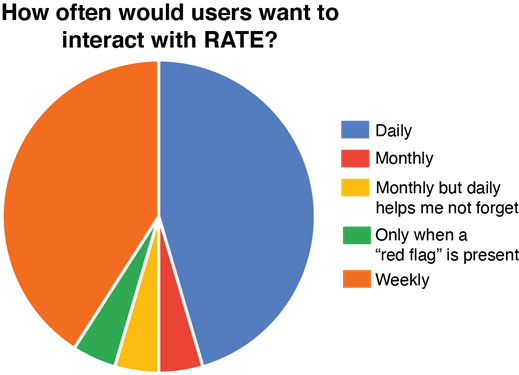


Supplementary Figure 3c: Survey results (Most responses were collected in March 2021). Principal investigators (24) commented about their preference for how often they would like to use the RATE UI for principal investigators (the "RATE Tracker Dashboard"). This question was asked within the context of an envisioned future version of the RATE UIs that alerts users to important changes with a 'push' interaction (something not included in the version used for this study). The actual survey question was, "How often would users want to interact with RATE?" From this context of a future 'push' capability, the question being asked could be more precisely described as, "If the RATE UI continuously alerted you to important changes, how often would you like to additionally open the RATE UI and review the information?" Results highlight a strong individual differences effect with about 86% of respondents indicating either "Daily" or "Weekly.


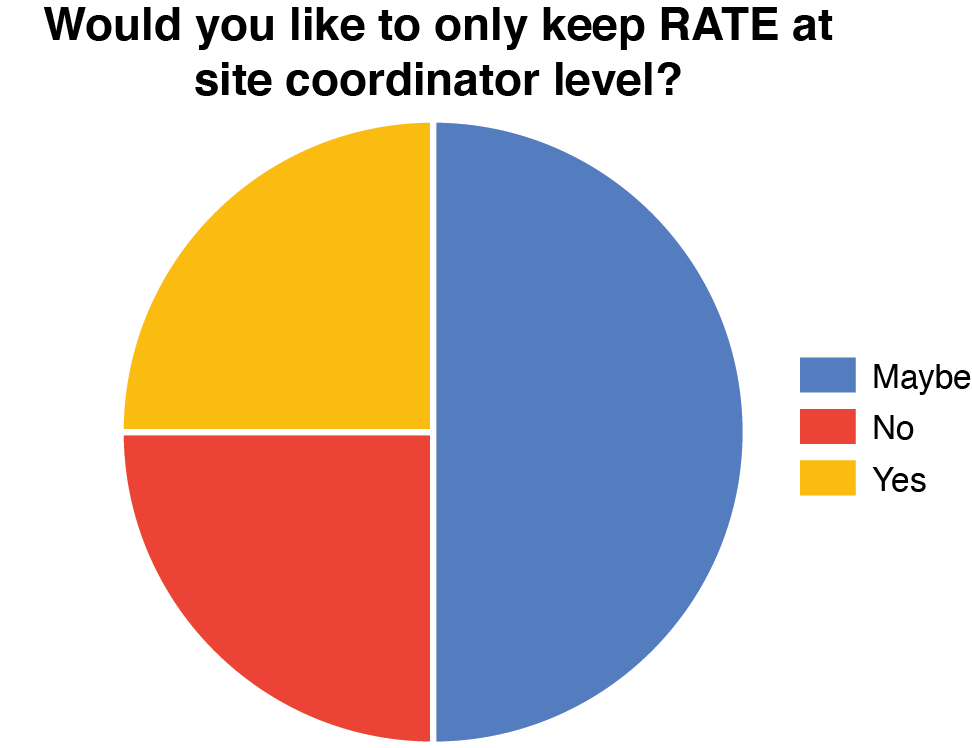


Supplementary Figure 3d: Survey results (Most responses were collected in March 2021). Principal investigators (24) commented about their preference for whether to enable individual military servicemembers to use the RATE UI (the "RATE Tracker" UI), as was the case for this study. The actual survey question was, "Would you like to only keep RATE at site coordinator level?" Results highlight a strong individual differences effect with disagreement on this question.


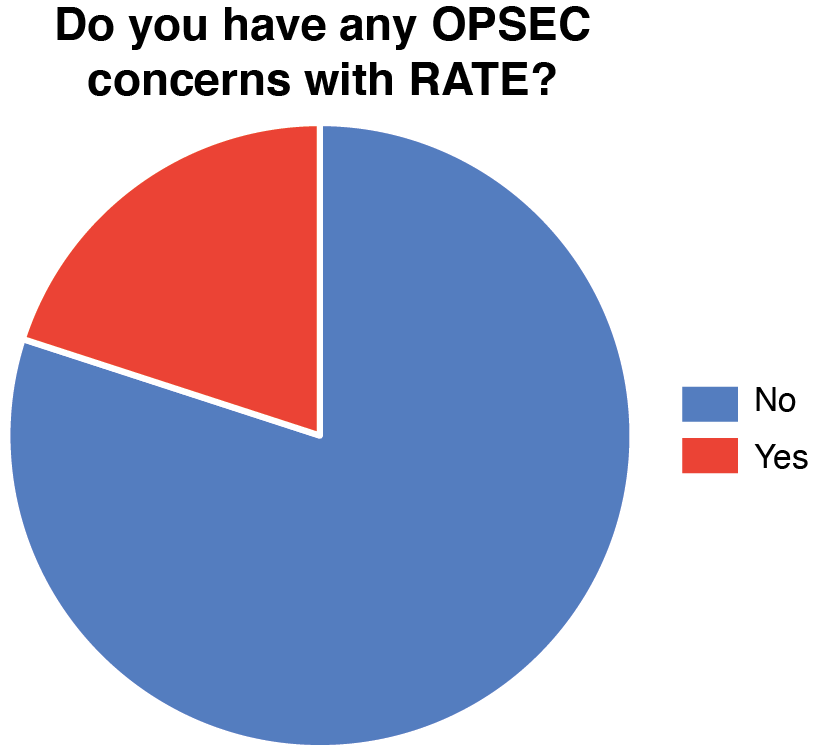
Supplementary Figure 3e: Survey results (Most responses were collected in March 2021). Principal investigators (24) commented about whether they had concerns about the operational security (OPSEC) relative to using the RATE service. The actual survey question was, "Do you have any OPSEC concerns with RATE?" Results highlight that most respondents (80%) had no OPSEC concerns for the use of RATE. This is a strong positive result given the US military's extreme sensitivities and requirements for high OPSEC.

References

38. Sharma, O., Sultan, A. A., Ding, H. & Triggle, C. R. A Review of the Progress and Challenges of Developing a Vaccine for COVID-19. *Front. Immunol.* **11**, 585354 (2020).

39. Mohan, S. V., Hemalatha, M., Kopperi, H., Ranjith, I. & Kumar, A. K. SARS-CoV-2 in environmental perspective: Occurrence, persistence, surveillance, inactivation and challenges. *Chem. Eng. J. Lausanne Switz. 1996* **405**, 126893 (2021).

40. Mooney, G. “A Menace to the Public Health” — Contact Tracing and the Limits of Persuasion. *N. Engl. J. Med.* **383**, 1806–1808 (2020).

41. Hashmi, H. A. S. & Asif, H. M. Early Detection and Assessment of Covid-19. *Front. Med.* **7**, (2020).

42. Koehlmoos, T. P., Janvrin, M. L., Korona-Bailey, J., Madsen, C. & Sturdivant, R. COVID-19 Self-Reported Symptom Tracking Programs in the United States: Framework Synthesis. *J. Med. Internet Res.* **22**, e23297 (2020).

43. Yu, F. *et al.* Quantitative Detection and Viral Load Analysis of SARS-CoV-2 in Infected Patients. *Clin. Infect. Dis. Off. Publ. Infect. Dis. Soc. Am.* **71**, 793–798 (2020).

44. Tang, Y.-W., Schmitz, J. E., Persing, D. H. & Stratton, C. W. Laboratory Diagnosis of COVID-19: Current Issues and Challenges. *J. Clin. Microbiol.* **58**, (2020).

45. Praharaj, I. *et al.* Pooled testing for COVID-19 diagnosis by real-time RT-PCR: A multi-site comparative evaluation of 5- & 10-sample pooling. *Indian J. Med. Res.* **152**, 88–94 (2020).

46. Toniolo-Barrios, M. & Pitt, L. Mindfulness and the challenges of working from home in times of crisis. *Bus. Horiz.* **64**, 189–197 (2021).

47. Quilty, B. J., Clifford, S., Flasche, S., Eggo, R. M., & CMMID nCoV working group. Effectiveness of airport screening at detecting travellers infected with novel coronavirus (2019-nCoV). *Euro Surveill. Bull. Eur. Sur Mal. Transm. Eur. Commun. Dis. Bull.* **25**, (2020).

48. Tirupathi, R., Bharathidasan, K., Palabindala, V., Salim, S. A. & Al-Tawfiq, J. A. Comprehensive review of mask utility and challenges during the COVID-19 pandemic. *Infez. Med.* **28**, 57–63 (2020).

49. Eykelbosh, A. *Physical Barriers for COVID-19 Infection Prevention and Control in Commercial Settings*. http://ncceh.ca/content/blog/physical-barriers-covid-19-infection-prevention-and-control-commercial-settings (2020) doi:10.13140/RG.2.2.32336.33283.

50. Chen, X. *et al.* Hand Hygiene, Mask-Wearing Behaviors and Its Associated Factors during the COVID-19 Epidemic: A Cross-Sectional Study among Primary School Students in Wuhan, China. *Int. J. Environ. Res. Public. Health* **17**, (2020).

51. Sum, Z. Z. & Ow, C. J. W. Community pharmacy response to infection control during COVID-19. A cross-sectional survey. *Res. Soc. Adm. Pharm.* **17**, 1845–1852 (2021).

52. Xie, W., Campbell, S. & Zhang, W. Working memory capacity predicts individual differences in social-distancing compliance during the COVID-19 pandemic in the United States. *Proc. Natl. Acad. Sci. U. S. A.* **117**, 17667–17674 (2020).

53. Baker, M. G., Peckham, T. K. & Seixas, N. S. Estimating the burden of United States workers exposed to infection or disease: A key factor in containing risk of COVID-19 infection. *PloS One* **15**, e0232452 (2020).

54. Ives, J. *et al.* Healthcare workers’ attitudes to working during pandemic influenza: a qualitative study. *BMC Public Health* **9**, 56 (2009).
